# An efficient churn prediction model using gradient boosting machine and metaheuristic optimization

**DOI:** 10.1038/s41598-023-41093-6

**Published:** 2023-09-02

**Authors:** Ibrahim AlShourbaji, Na Helian, Yi Sun, Abdelazim G. Hussien, Laith Abualigah, Bushra Elnaim

**Affiliations:** 1 Department of Computer Science, University of Hertfordshire, Hatfield, UK; 2 Department of Computer and Network Engineering, Jazan University, 82822-6649 Jazan, Saudi Arabia; 3 Department of Computer and Information Science, Linköping University, Linköping, Sweden; 4 Faculty of Science, Fayoum University, Faiyum, Egypt; 5 Computer Science Department, Prince Hussein Bin Abdullah Faculty for Information Technology, Al Al-Bayt University, Mafraq, 25113 Jordan; 6 Department of Electrical and Computer Engineering, Lebanese American University, 13-5053 Byblos, Lebanon; 7 Hourani Center for Applied Scientific Research, Al-Ahliyya Amman University, Amman, 19328 Jordan; 8 MEU Research Unit, Middle East University, Amman, 11831 Jordan; 9 Applied Science Research Center, Applied Science Private University, Amman, 11931 Jordan; 10 School of Computer Sciences, Universiti Sains Malaysia, 11800 Pulau Pinang, Malaysia; 11 School of Engineering and Technology, Sunway University Malaysia, 27500 Petaling Jaya, Malaysia; 12 Department of Computer Science, College of Science and Humanities in Al-Sulail, Prince Sattam Bin Abdulaziz University, 11671 Riyadh, Saudi Arabia

**Keywords:** Biomedical engineering, Computer science, Software

## Abstract

Customer churn remains a critical challenge in telecommunications, necessitating effective churn prediction (CP) methodologies. This paper introduces the Enhanced Gradient Boosting Model (EGBM), which uses a Support Vector Machine with a Radial Basis Function kernel (SVM_RBF_) as a base learner and exponential loss function to enhance the learning process of the GBM. The novel base learner significantly improves the initial classification performance of the traditional GBM and achieves enhanced performance in CP-EGBM after multiple boosting stages by utilizing state-of-the-art decision tree learners. Further, a modified version of Particle Swarm Optimization (PSO) using the consumption operator of the Artificial Ecosystem Optimization (AEO) method to prevent premature convergence of the PSO in the local optima is developed to tune the hyper-parameters of the CP-EGBM effectively. Seven open-source CP datasets are used to evaluate the performance of the developed CP-EGBM model using several quantitative evaluation metrics. The results showed that the CP-EGBM is significantly better than GBM and SVM models. Results are statistically validated using the Friedman ranking test. The proposed CP-EGBM is also compared with recently reported models in the literature. Comparative analysis with state-of-the-art models showcases CP-EGBM's promising improvements, making it a robust and effective solution for churn prediction in the telecommunications industry.

## Introduction

Customers are the most valuable resource as they present the main reason for any industry's success. On the other hand, a churner is a customer who abandons his current company to join another competing company's service in the market. Customer churn is a common problem in the telecom business, and companies in this sector try to minimize churn rates. Studies show that reducing the customer churn rate saves money, as acquiring a new customer costs five times more than satisfying an existing one^1^. Therefore, reducing the churn rate has become particularly important for preserving revenues in this sector. Because of the significant financial implications of correctly predicting customer churn, CP models have become vital in CRM to identify customers most likely to terminate their relationships. As a result, there has been much focus on developing new methods to improve the accuracy of the CP using ML.

Nowadays, ML techniques are used to predict future patterns and behaviors of customers^2^, so marketing strategies can be improved according to the produced results from these models. ML approaches can play a critical part in the success of different applications, such as oil price prediction^3^, sentiment analysis^4^, energy consumption^5^, medical diagnosis^6^, and CP^7^. These applications use one type of ML family of algorithms, called ensemble methods, which are inspirited by the human cognitive system. These methods have the powerful capability to deal with high-dimensional data and generate several diverse solutions for a given task^8^.

Ensemble methods build many base models and then merge them into one to achieve better prediction results than using a single base model. Bagging and boosting are the most popular ensemble methods^9^. The bagging method, also known as "bootstrap aggregation," is based on averaging the base models, while the boosting methods are built upon a constructive iterative mechanism. In boosting algorithms, several weak learners are combined stage-wise to obtain a strong learner with improved prediction accuracy^10^. The family of boosting methods depends on different constructive strategies of ensemble formation. A gradient-descent-based formulation of boosting methods, called Gradient Boosting Machine (GBM), is derived by^11^. The GBM can be considered an optimization model aiming to train a series of weak-learner models, which sequentially minimizes a pre-defined loss function.

According to^12^, several essential choices of differentiable weak-learner models and loss functions can be customized to a given task in the GBM model, making this model highly flexible to be applied in several ML applications based on the task requirements^13–15^. This paper aims to develop a new model by improving GBM's structure to effectively predict customer churn in the telecom sector. The main contributions of this paper can be summarized as follows:

- CP-EGBM is a new model with high predictive performance that may be used to develop effective strategies and contains customer churn risks in the telecom sector. It can enhance the learning ability of the GBM model structure by using SVM as a base learner and exponential loss as a loss function.
- Boosting the capability of the PSO in the exploration phase using the consumption operator of the AEO method could effectively find the most suitable values of the CP-EGBM's hyper-parameters.
- The performance of the proposed CP-EGBM is assessed using seven datasets in several evaluation metrics.
- The CP-EGBM model outperformed either GBM or SVM alone, and it is superior to several earlier reported models in the literature, making it more suitable for CP.

The rest of this paper is arranged as follows. “Literature review” provides a literature review on CP. The proposed CP-EGBM model is presented in “Proposed CP-EGBM”, and “Experimental results” discusses the experimental results. Lastly, the conclusions of this paper and possible future works are provided in “Conclusion and future works”.

## Literature review

Many works applied ensemble ML models to predict customer churn^7,16,17^. Wang et al*.*^18^ investigated the capability of the GBM model for CP. They used a large customer dataset obtained from the Bing-Ads platform company to identify whether the customers would leave or stay based on the analysis of their historical data records. The results showed that GBM was an effective and efficient model for predicting churner customers in the near future.

Several comparative analyses are conducted for CP using ML models. Ahmad et al*.*^19^ compared four ML models, including Decision Trees (DTs), Random Forest (RF), GBM, and Extreme Gradient Boosting (XGBoost), for customer churn prediction. The results showed that the XGboost method outperformed other models when they evaluated the models using big data provided by a telecom company in Syria. Jain et al*.*^20^ used four models for CP in the banking, telecom, and IT sectors, where they used Logistic Regression (LR), RF, SVM, and XGBoost. The results showed that XGBoost performed better than others in the telecom sector. In another work, Dhini et al*.*^21^ compared RF and XGBoost to find the best model for CP. They used a private dataset collected from different companies in Indonesia to evaluate the models. The results showed that the predictive performance of the XGBoost was better than that of the RF model. In Sabbeh^22^, the author compared a set of ML models using a publicly available dataset for CP. The results showed that RF attained the best results compared to other models used in their work.

Sandhya et al*.*^23^ applied LR, K-Nearest Neighbors (KNN), SVM, and RF models to a publicly available dataset for CP. The authors first preprocessed the dataset and overcame the class imbalance problem using Synthetic Minority Oversampling Technique (SMOTE). The obtained results showed that RF performed better than the other models. Kimura^24^ used six ML models: LR, RF, SVM, CatBoost, XGBoost and LightGBM. For data preprocessing, the authors used SMOTE Tomek Link and SMOTE-ENN sampling methods to balance class distribution in a publicly available dataset for CP. The results showed that CatBoost with SMOTE is the best model. Zhu & Liu^25^ conducted a comparative study between ten ML models for churn prediction using a publicly available dataset; the results indicated that XGBoost obtained the best accuracy compared to the other models.

Kanwal et al*.*^26^ employed a hybrid CP model using PSO to select the most informative features in a publicly available dataset for CP. Then, the selected features are used as inputs to DTs, KNN, Gradient Boosted Tree (GBT), and NB models. The findings indicate that the PSO with the GBT model obtained successful accuracy outcomes compared to the other models. Bilal et al*.*^27^ introduced a CP model based on hybrid clustering and classification methods to predict customer churn from two publicly available datasets. The results showed that this model is more robust than the other existing models in the literature.

The stacking model technique (i.e., a mechanism that aims to leverage the benefits of a set of base models while ignoring their disadvantages) is also used for CP. Karuppaiah & Gopalan^28^ presented a stacked Customer Lifetime Value-based heuristic incorporated ensemble model to predict customer churn. The authors used a publicly available dataset to evaluate the proposed model, and the obtained accuracy results showed good performance compared to the other existing models in the literature. Rabbah et al*.*^29^ proposed a new CP model using deep learning and stacked models. They used a publicly available dataset to validate their model; the dataset is first preprocessed and balanced by the SMOTE method and then used a pre-trained Convolutional Neural Network (CNN) to select the essential features from the dataset. They employed the stacking model technique (i.e., a mechanism that aims to leverage the benefits of a set of base models while ignoring their disadvantages) to predict customer churn. The results demonstrated high efficacy of the developed model than the DTs, LR, RF, XGBoost, and Naive Bayes (NB) models.

Karamollaoglu et al*.*^30^ used to separate datasets for CP in the telecommunication industry. Eight ML models are explored, including LR, KNN, DT, RF, SVM, AdaBoost, NB, and multi-layer perceptron. Although all models reported good performance, ensemble-based RF models showed the highest performance. Akinrotimi et al*.*^31^ used oversampling techniques for class imbalance problems and applied the dimensionality reduction technique to pick out optimal features with strong predictive ability. They used LR and the NB models as classification strategies for CP. The results showed that NB provided more efficient results than LR. Akbar and Apriono^32^ used XGBoost, Bernoulli NB, and DT models for CP and showed that XGBoost attained the best performance compared to other models.

Based on the provided research works on customer CP, the following research gaps can be identified:

- Limited exploration of ensemble models: while some studies have applied ensemble models for CP, such as stacking models, there is still a need for further exploration and evaluation of different ensemble techniques and their effectiveness in improving prediction accuracy.
- Limited investigation of hybrid models: hybrid models that combine different machine learning algorithms or feature selection techniques have shown promising results in CP. However, there is still a lack of comprehensive studies comparing various hybrid models and evaluating their performance on different datasets.
- Lack of focus on industry-specific CP: many studies have evaluated CP models on publicly available datasets, but there is a need for more research focusing on specific industries, such as banking, telecom, and IT. Different industries may have unique characteristics and churn patterns, requiring customized CP approaches.
- Preliminary analysis of feature selection techniques: feature selection plays a crucial role in CP, as it helps identify the most informative features for accurate prediction. However, the existing literature lacks comprehensive analyses and comparisons of different feature selection techniques and their impact on CP performance.
- Lack of comparison across multiple performance metrics: many studies focus on a single performance metric, such as accuracy or F1-measure, for evaluating CP models. However, a comprehensive comparison across multiple metrics, including precision, recall, and area under the receiver operating characteristic curve (AUC-ROC), is essential to understand different models overall performance and effectiveness.

Addressing these research gaps would contribute to advancing the field of customer churn prediction by providing insights into the effectiveness of different models, techniques, and approaches in various industry contexts and facilitating more accurate and proactive customer retention strategies. Although existing models based on ensemble methods achieved tremendous success in the application of CP, there is still a need for more efforts to provide this sector with an efficient and accurate model which can identify churner and non-churner customers accurately and can assess decision-makers in this sector to develop more effective strategies in order to reduce customer churn rate. The GBM model shows excellent potential in classification problems. It typically uses a DT as a base learner to initialize the model, which is sub-optimum^12^. SVM is a powerful mathematical model that proves its ability to solve CP problems^23,30^. Choosing an effective base learner as a starting point for the GBM learning process could produce an effective GBM model. Hence, in this work, the base- learner in the GBM is replaced with the SVM. In addition, the hyper-parameters for the modified GBM are optimized using a modified version of the PSO method. To the best knowledge of the authors, optimizing GBM has never been applied in CP so far. This paper presents a new model that improves the GBM structure and optimizes its hyperparameters to predict customer churn effectively. The proposed model can assess improving CP's efficiency and designing optimal decisions and policies in this sector.

## Proposed CP-EGBM

**Figure 1:**
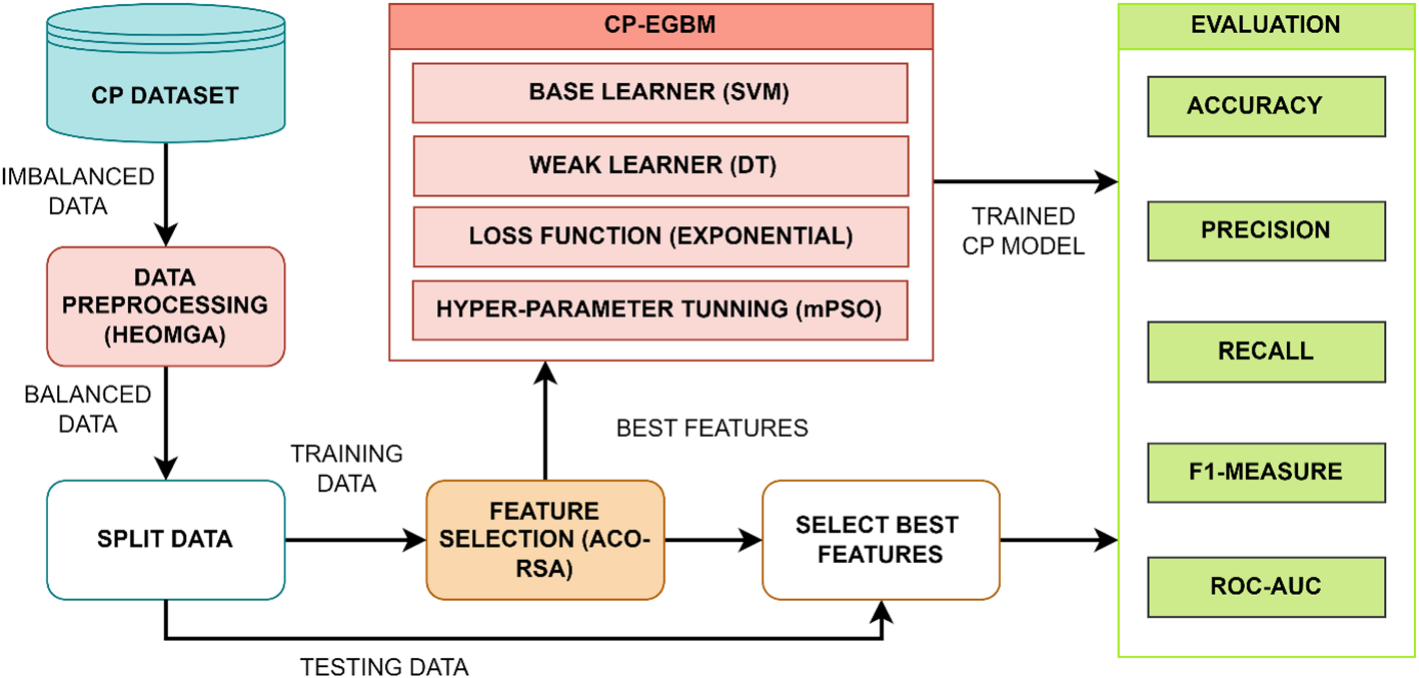
Flowchart of the proposed CP with the enhanced GBM (EGBM) model.

The overall process flow of our CP is depicted in Fig. 1, with the proposed CP-EGBM classification model in red. The following sub-sections provide the details of the model.

### Data preprocessing and feature selection

Let the dataset consist of *N* examples of *M*-dimension feature vectors $\left\{{x}_{n,m}, 1\le n\le N \mathrm{and} 1\le m\le M\right\}$ and target label $\left\{y, 1\le y\le C\right\}$ where *C* is the number of classes. Each feature in the dataset is normalized in the range [0, 1] as per Eq. (1) to improve classification capability.

1 $${\widehat{x}}_{n,m}=\frac{{x}_{n,j}-{x}_{j}^{min}}{{x}_{j}^{max}-{x}_{j}^{min}}$$

where, ${x}_{j}^{min}$ and ${x}_{j}^{max}$ are minimum and maximum values for the *j*th feature dimension and ${\widehat{x}}_{i,j}$ is the normalized value of *j*th feature for *i*th example.

The performance of most ML models degrades for a class-imbalanced dataset. A dataset balance can be checked by comparing the number of examples for each class label $y$. For balancing the dataset, the minority class examples are oversampled to match the number of examples using the Heterogeneous Euclidean-Overlap Metric Genetic Algorithm (HEOMGA) approach^33^.

Another critical factor affecting the performance of ML models is the input feature dimensional space. The significant features for classification are selected from the normalized-balanced dataset using Ant Colony Optimization- Reptile Search Algorithm (ACO-RSA) approach^34^. The ACO-RSA is a recent Meta-Heuristic (MH) approach published as a feature selection method for CP. The optimal feature set comprises only the most significant features for classification. Finally, the datasets are split into two exclusive and exhaustive sets for training and testing the proposed CP-EGBM model.

### Classification using CP-EGBM

An overview of the GBM, a description of the developed CP-EGBM, and Hyper-parameter optimization for the CP-EGBM are given in this section.

#### Gradient boosting machine (GBM)

Gradient Boosting Machine (GBM)^11^ combines a set of weak learners by focusing on the resulting error at each iteration until a strong learner is obtained as a sum of the successive weak ones.

Let $D= {\left\{{x}_{n},{y}_{n}\right\}}_{n=1}^{N}$ denote training examples where the goal of gradient boosting is to find an optimal estimate $F\left(x\right)$ of an approximation function ${F}^{*}$(x), which maps the instances ${x}_{n}$ to ${y}_{n}$ to minimize the expected value of a given loss function $L (y, F(x))$ over the distribution of all training examples.

2 $${F}^{*}(\mathrm{x})={\mathrm{argmin}}_{F\left(x\right)}{L}_{x,y}\left(y, F\left(x\right)\right)$$

GBM uses a logistic loss function for classification tasks to estimate approximation function $L\left(y, F\left(x\right)\right)={\left(y-F\left(x\right)\right)}^{2}$^35^. GBM starts with a weak learner $F\left(x\right)$ that is usually a constant value, and then it fits each weak learner to correct the errors made by the previous weak learner to strengthen prediction performance by minimizing loss function over each boosting stage^36^. At each stage, the local minimum proportional takes steps to the loss function's negative gradient to find the local minimum. The gradient direction of the loss function at *i*th boosting stage can be calculated as

3 $${r}_{i,n}={- \left\lceil\frac{\partial L\left({y}_{n}, F\left({x}_{n}\right)\right)}{\partial F\left({x}_{n}\right)} \right \rceil}_{F\left(x\right)={F}_{i-1}\left(x\right)}$$

GBM generalizes the calculation range of the gradient when regression trees is used with parameter $a$ as weak-learners, usually a parameterized function of the input variables $x$, characterized by the parameters $a$ and $\partial$ indicates the partial derivative. The tree can be obtained by solving the following:

4 $${a}_{i}={\mathrm{argmin}}_{a,\beta }{\sum }_{n=1}^{N}{\lceil{r}_{i,n}-\beta h\left({x}_{n}, a\right)\rceil}^{2}$$

where, ${a}_{i}$ is a parameter that is obtained at iteration $i$, and $\beta$ is the weight value (i.e., the expansion coefficient of the weak learner). Then the optimal length ${p}_{i}$ is determined, and the model ${F}_{i}\left(x\right)$ is updated at each iteration *i*, with t = 1 to the number of iterations T, as in steps 5 and 6 below in the GBM algorithm. GBM is detailed in Algorithm 1^11^.


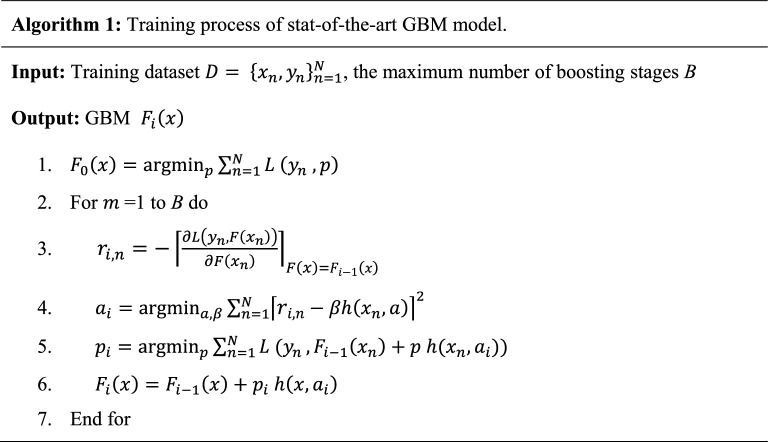


The choices of base learners and loss functions derived from the GBM model facilitate the capacity to design and further development in this model by researchers based on the task requirements^11,12^. This work aims to develop a new classification model for the application of CP by enhancing the structure of the GBM and its hyper-parameters, as will be discussed in the following subsections.

#### Develop CP-EGBM

As mentioned earlier, the GBM model typically uses a DT as the base learner. At each boosting stage, a new DT (weak learner) is fitted to the current residual and concatenated to the previous model to update the residual. This process continues until the maximum number of boosting stages is reached^12^. However, using DT as a base learner might not optimally approximate a smooth function since DT extrapolates the relationship between the input/output data points with a constant value^5^. Thus, using a DT to start the GBM model training process could result in poor predictive performance and overfitting.

In the GBM model, various base-learners are derived, divided into linear, smooth, and DTs models^12^, and optimized the GBM using different manners^37–39^. However, no previous works focused on changing the base learner of the GBM to improve its structure using the SVM in CP. The SVM model introduced in^40^ proves its ability to solve various classification problems^41^. As for most classifiers, SVM depends on the training data to build its model by finding the best decision hyperplane that separates the class labels (i.e., response variables). The main goal of the SVM is to find the optimum hyperplane by maximizing the margin and minimizing classification error between each class. In addition, using kernel functions strategy and its applicability to the linearly non-separable data can be extended to map input data into a higher dimensional space. The hyperplane can be described as follows^42^:

5 $$\mathrm{w}.{x}_{i}+\mathrm{b}=0,$$

where, $\mathrm{w}$ is an average vector, and $\mathrm{b}$ is the position of the relative area to the coordinate center.

The optimization of the margin to its support vectors can be converted into a constrained programming problem as:

6 $$\mathrm{min}{\frac{1}{2}[\![w]\!]}^{2}+C\sum_{i=1}^{N}{\zeta }_{i} \text{ s.t.} {y}_{i}\left({w}^{T}{x}_{i}+b\right)\ge 1- {\zeta }_{i}\text{ and }{\zeta }_{i}\ge 0$$

where, ${\zeta }_{i}$ represents the misclassified samples to the corresponding margin hyperplane, and $C$ is the cost of the penalty.

The SVM model's most widely used kernel functions are Linear, Polynomial, Sigmoid, and Radial Basis functions (RBF). Among them, RBF is preferable due to its reliability in implementation, adaptability to handle very complex parameters and simplicity^42^. In this research, the SVM with RBF kernel (SVM_RBF_) is integrated as a base learner in the GBM's structure to boost its learning capability and provide a more accurate approximation of the target label. The RBF kernel function can be given as:

7 $$k\left({\mathrm{x}}_{\mathrm{n}} ,{\mathrm{x}}_{\mathrm{i}}\right)=\mathrm{exp}\left(-\upgamma {\Vert {\mathrm{x}}_{\mathrm{n}}-{\mathrm{x}}_{\mathrm{i}}\Vert }^{2}+C\right)$$

where ${\mathrm{x}}_{\mathrm{n}} ,{\mathrm{x}}_{\mathrm{i}}$ are vectors of features computing from training or test data points, $\gamma$ determines the influence of each training example, and C is the cost or penalty.

The GBM learning performance for a given task depends greatly on the loss function^12,36^. Therefore, it is essential to carefully select the loss function and the function to calculate the corresponding negative gradients in the GBM model's structure. Several loss functions are reported in the literature for classification, including logistic regression (i.e., deviance), Bernoulli, and exponential. A comparison of loss functions is presented in the next section. The pseudo-code of the developed CP-EGBM is given in Algorithm 2.
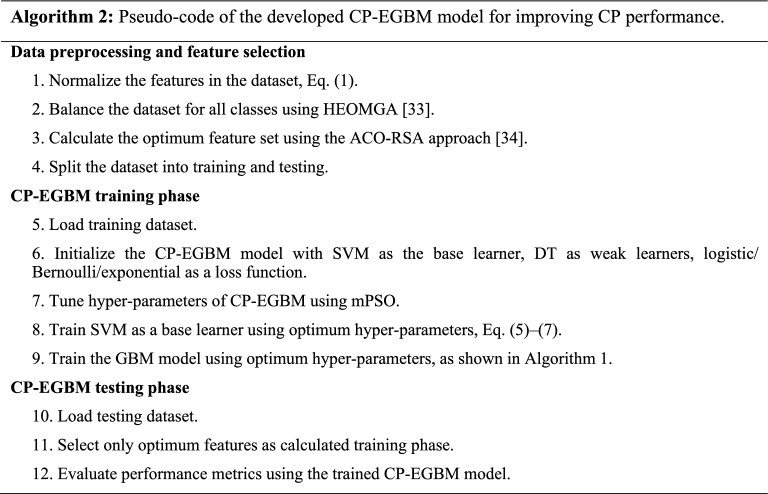


#### Hyper-parameter optimization

Parameter setting is essential in enhancing the models' efficacy and performance. Traditionally, hyper-parameters can be selected using a trial-and-error. However, manually tuning the parameters is often time-consuming, yielding unsatisfactory results without deep expertise. MH method can tune the model's hyper-parameters for solving this problem. Two MH methods, PSO and AEO, are presented in the following subsections, and the modified PSO (mPSO) method is introduced.

##### Particle swarm optimization (PSO)

PSO is an MH method inspired to simulate the social and group behaviors of animals, humans, and insects^43^. This method uses a set of particles (initial population) to traverse a given search space randomly. In each iteration, the position of each particle $x$ and the velocity $\upsilon$ of this particle are updated using the best position in the current population.

Let there be *P* particles in the *K*-dimensional search space. The position $x(t)$ and velocity $\upsilon (t)$ at the time of *t* are expressed as:

$${x}_{i}\left(t\right)={\left[{x}_{i1}\left(t\right),{x}_{i2}\left(t\right)\cdots {x}_{iK}\left(t\right)\right]}^{T} \mathrm{ for } 1\le i\le P$$

8 $${\upupsilon }_{i}\left(t\right)={\left[{\upupsilon }_{i1}\left(t\right),{\upupsilon }_{i2}\left(t\right)\cdots {\upupsilon }_{iK}\left(t\right)\right]}^{T}$$

The fitness, the local best position ${P}_{best}$ and global best position ${G}_{best}$ at time *t* are represented as:

$${P}_{best}\left(t\right)={\left[{\mathrm{P}}_{1}\left(t\right),{\mathrm{P}}_{2}\left(t\right)\cdots {\mathrm{P}}_{K}\left(t\right)\right]}^{T}$$

9 $${G}_{best}\left(t\right)={\left[{\mathrm{G}}_{1}\left(t\right),{\mathrm{G}}_{2}\left(t\right)\cdots {\mathrm{G}}_{K}\left(t\right)\right]}^{T}$$

At time $t+1$, the velocity $\upsilon (t+1)$ of the particle is updated as,

10 $${\upupsilon }_{i}\left(t+1\right)=w{\upupsilon }_{i}\left(t\right)+{c}_{1}{r}_{1}\left({\mathrm{P}}_{besti}\left(t\right)-{\mathrm{x}}_{1}\left(t\right)\right)+{c}_{2}{r}_{2}\left({G}_{best}\left(t\right)-{x}_{i}\left(t\right)\right)$$

where $w$ is an inertia weight factor that controls the velocity and allows the swarm to converge, ${c}_{1}$ is the cognitive factor and ${c}_{2}$ is the social factor that controls the randomness added to the velocity $\upsilon (t+1)$ for the next position ${x}_{i}(t+1)$, ${r}_{1}$ and ${r}_{2}$ are two random vectors in the range [0,1].

11 $${x}_{i}\left(t+1\right)={x}_{i}\left(t\right)+{\upsilon }_{i}\left(t+1\right)$$

where the next position ${x}_{i}\left(t+1\right)$ of *i*th particle is computed using the current position ${x}_{i}\left(t\right)$ and updated velocity ${\upsilon }_{i}\left(t+1\right)$ as generated in Eq. (10). Finally, ${x}_{i}$ vectors present solutions while ${\upupsilon }_{i}$ presents the momentum of particles.

##### Artificial ecosystem-based optimization (AEO)

AEO is another MH method motivated by the energy flow in the natural ecosystem, introduced by^44^. AEO uses three operators to achieve optimal solutions, as described below.

Production
  In this operator, the producer represents the worst individual in the population. Thus, it must be updated concerning the best individual by considering the upper and lower boundaries of the given search space so that it can guide other individuals to search other regions. The operator generates a new individual between the best individual ${x}_{best}$ (based on fitness) and the randomly produced position of individuals in the search space ${x}_{r\mathrm{a}nd}$ by replacing the previous one. This operator can be given as,
  12 $${x}_{i}\left(t+1\right)= \left(1-\alpha \right){x}_{best}\left(t\right)+{\alpha x}_{rand}\left(t\right)$$
  13 $$\alpha =(1-t/\mathrm{T}){r}_{1}$$
  14 $${x}_{rand}={r}_{2}\left(UB-LB\right)+LB$$
  where ${x}_{rand}\left(t\right)$ guides the other individuals to explore search space in the subsequent iterations broadly, ${x}_{i}\left(t+1\right)$ leads the other individuals to exploitation in a region around ${x}_{best}\left(t\right)$ intensively, $\alpha$ is a linear weight coefficient to move the individual linearly from a random position to the position of the best individual ${x}_{best}\left(t\right)$ through the pre-defined maximum number of iterations *T*, ${r}_{1}$ and ${r}_{2}$ are random numbers in the interval [0, 1], and $UB$ and $LB$ represent the upper and lower boundaries of the search space.
2. Consumption
  This operator starts after the production operator is completed. It may eat a randomly chosen low-energy consumer, a producer, or both to obtain food energy. A Levy flight-like random walk, called Consumption Factor (CF), is employed to enhance exploration capability, and it is defined as follows:
  15 $$\mathrm{CF}=\frac{1}{2}\frac{{v}_{1}}{\left|{v}_{2}\right|}, \quad {v}_{1},{v}_{2}\in N\left(\mathrm{0,1}\right)$$
  where, $N\left(\mathrm{0,1}\right)$ is a normal distribution with zero mean and unity standard deviation.
  Different types of consumers adopt different consumption behaviors to update their positions. These strategies include:
  Herbivore behavior: a herbivore consumer would eat only the producer and can be formulated as:
    16 $${x}_{i}\left(t+1\right)={x}_{i}\left(t\right)+CF.\left({x}_{i}\left(t\right)-{x}_{1}\left(t\right)\right), \quad i\in \left[2,\dots P\right]$$
  Carnivore behavior: A carnivore consumer would only eat another consumer with higher energy, and it can be modeled as:
    17 $${x}_{i}\left(t+1\right)={x}_{i}\left(t\right)+CF.\left({x}_{i}\left(t\right)-{x}_{r\mathrm{a}nd\in \left(0, 2i-1\right)}\left(t\right)\right), \quad i\in \left[3,\dots P\right]$$
  Omnivore behavior: An omnivore consumer can eat a random producer or a producer with higher energy, and this behavior can be formulated as:
    18 $${x}_{i}\left(t+1\right)={x}_{i}\left(t\right)+CF\left({r}_{2}{(x}_{i}\left(t\right)-{x}_{1}\left(t\right))\right)+(1-{r}_{2}){(x}_{i}\left(t\right)-{x}_{r\mathrm{a}nd\in \left(0, 2i-1\right)}\left(t\right)), \quad i\in \left[3,\dots P\right]$$

3. Decomposition
  In this final phase, the ecosystem agent dissolves. The decomposer breaks down the remains of dead individuals to provide the required growth nutrients for producers. The decomposition operator can be expressed as:
  $${x}_{i}\left(t+1\right)={x}_{P}\left(t\right)+De(e{ . x}_{P}\left(t\right)-h.{x}_{\mathrm{ra}nd\in \left(0, 2i-1\right)}\left(t\right)), i\in \left[1,\dots P\right]$$
  19 $$\mathrm{where } \; De=3u \quad u\in N\left(0, 1\right), e={r}_{3} . randi\left(\left[1, 2\right]\right)- 1, \mathrm{and} \; h=2{r}_{3}-1$$
  and $e$, $h$, and $De$, are weight coefficients designed to model decomposition behavior.

##### Modified PSO (mPSO) method

The exploration phase is integral to MH algorithms, aiming to find better solutions by investigating search space. PSO suffers from premature convergence to a local minimum, which makes it spend most of the time on locally optimal solutions. Hence, it is weak in exploring new areas in the search space^45,46^.

A modified PSO (mPSO) method aims to avoid premature convergence in the local optima and, thus, enhance its capability to tune optimum hyper-parameters for the CP-EGBM model. The mPSO method integrates the consumption operator of the AEO into the PSO method's structure. As discussed in the previous subsection, the consumption phase in the AEO method is responsible for exploration, and it has three leading operators: Herbivore, Carnivore, and Omnivore. Both herbivores and omnivores are based on the producer solution (i.e., equals to the best solution in the swarm); the last operator depends on two randomly selected solutions, which helps explore new regions in the search space. The mPSO method utilizes the strength of the AEO in exploration (Eq. 15) and the strength of the PSO in exploitation (Eq. 10) to select optimum hyper-parameters for the CP-EGBM model. The mPSO can be presented as (Eq. 20): The pseudo-code of the mPSO is described in Algorithm 3.

20 $${\upsilon }_{i}\left(t+1\right)=w{\upsilon }_{i}\left(t\right)+{c}_{1}{r}_{1}\left(CF-{x}_{1}\left(t\right)\right)+{c}_{2}{r}_{2}\left({G}_{best}\left(t\right)-{x}_{i}\left(t\right)\right)$$

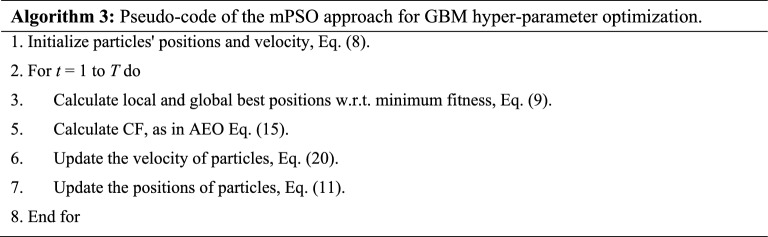


#### Evaluation measures

In this study, the CP-EGBM model is assessed using a set of evaluation measures, including, Accuracy, Precision, Recall, F1-measure, and Area under the ROC Curve (AUC), and they are computed as follows:

21 $$AC= \frac{TP+TN}{TP+TN+FN+FP}$$

22 $${\text{Recall}} (R)= \frac{TP}{TP+FN}$$

23 $$\text{F1-measure} (F)= \frac{\left(TP+FN\right)\left(TP+FP\right)}{TP\left(2TP+FN+FP\right)}$$

24 $$\mathrm{AUC}= \frac{1}{2}\left(1+ \frac{TP}{TP+FN}- \frac{FP}{FP+TN}\right)$$

where True Positive and (TP) and True Negative (TN) denote the correctly detected samples as positive and negative, respectively; similarly, False Negative (FN) and False Positive (FP) represent the number of misclassified positive and negative examples.

## Experimental results

The experiments performed to assess the CP-EGBM model, comparing its performance with the GBM and SVM_RBF_ models, are described.

### Experimental setup

The performance of the CP-EGBM is validated by conducting experiments on publicly available datasets for CP. The characteristics of these Datasets (DSs) are presented in Table 1. The HEOMGA^33^ is used for data balancing and ACO-RSA^34^ is employed for FS on all the datasets. Possible bias in selecting the training and testing datasets is avoided using the tenfold cross-validation (CV) technique is employed. All the experiments are implemented using Python and executed on a 3.13 GHz PC with 16 GB RAM and Windows 10 operating system.

**Table 1:** Characteristics of the open-source CP datasets used for evaluating the developed CP-EGBM.

Dataset description	DS 1	DS 2	DS 3	DS 4	DS 5	DS 6	DS 7
# of instances	3333	7043	71,047	100,000	3333	3150	50,375
# of features	21	21	58	100	11	16	10
# of class	2	2	2	2	2	2	2
Source	^31,32^	^31,32^	^32^	^31,32^	^32^	^32^	^32^

### Base learner and its behavior in the GBM model

**Figure 2:**
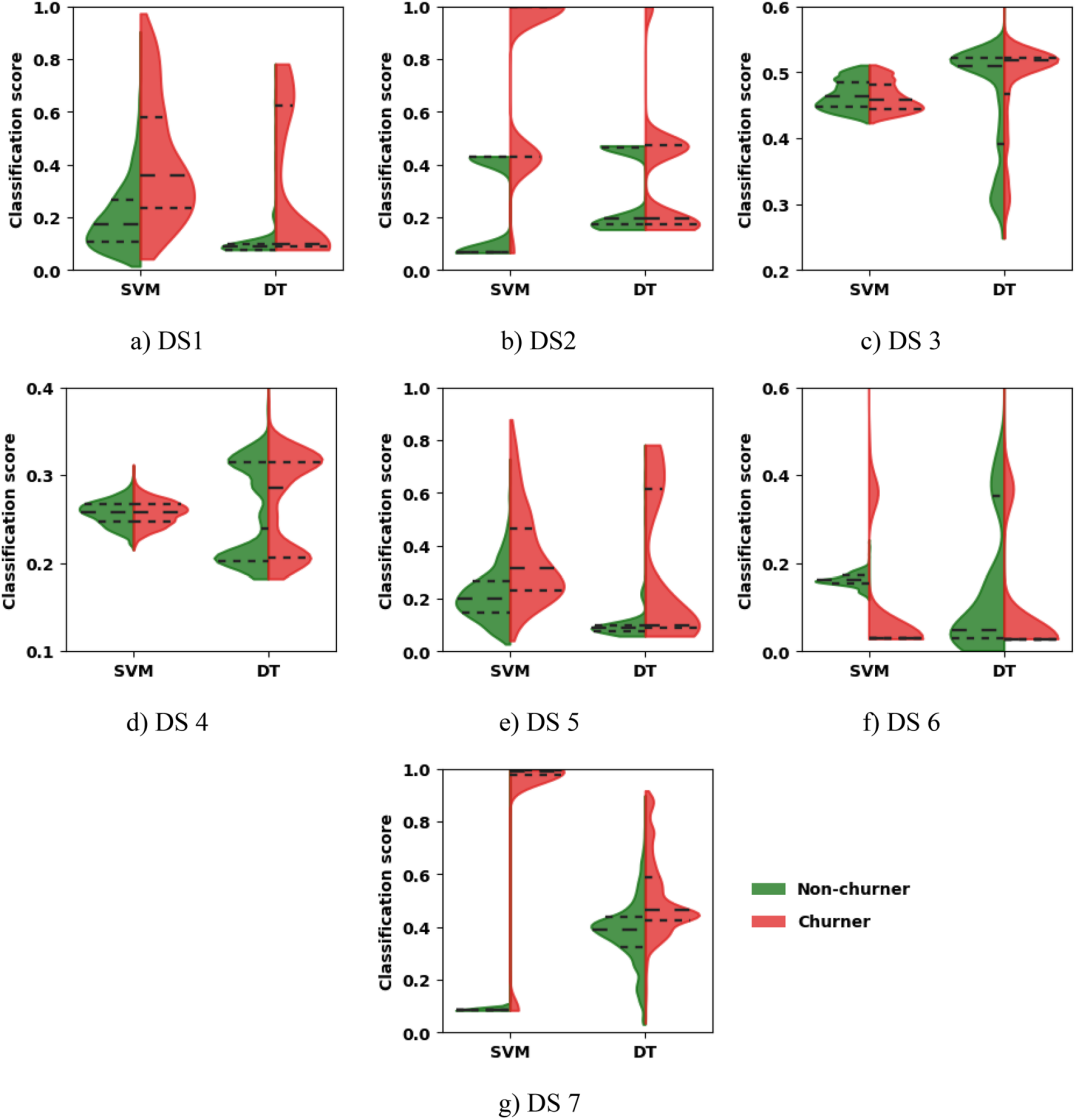
Comparative analysis of testing dataset classification scores generated by SVM_RBF_ and DT base learners based on probability density distribution of non-churners and churners in all the datasets.

To examine the effect of changing the base learner from DT to SVM_RBF_ in the GBM model, Probability Density Distribution is used, and the test dataset classification score (which is a number between '0' and '1', indicating the degree how much a testing example belongs to Churner/Non-churner class) generated by both base learners are visualized using the Violin plot method^47^, as shown in Fig. 2. A classification score is a raw continuous-valued probabilistic output of the ML model. For binary classification, one class (assume churner) has a classification score $p$ then another class will have a score $1-p$ .

The Violin plot is a method similar to the box plot with an additional characteristic called probability density, typically smoothed by a kernel density estimator. An interquartile range is calculated for each distribution to compare base learners' dispersion of non-churner and churner classes. The horizontal dotted lines in each class group indicate the first (25th percentile of the data), the second (50th percentile of the data or median), and the third (75th percentile of the data) quartiles to the corresponding distribution. The similarity/closeness of the two distributions is directly proportional to the closeness of these quartiles.

The visualization in Fig. 2 shows that the quartiles of classification score using SVM_RBF_ as a base learner in DS 1, DS 2, DS 5, DS 6, and DS 7 well-separate churners (in red) and non-churners (in green) than the quartiles using the DT. Using SVM_RBF_ as a base learner better classifies the Churner and Non-churner than DT. In DS 3 and DS 4, distributions for churners and non-churners are similar for both base learners, also indicated by closer quartiles for both classes, resulting in poor classification for both base learners. These results confirm and prove the suitability of the SVM_RBF_ to be used as a base learner in the developed CP-EGBM model.

### Loss function selection

The loss function gives a general picture of how well the model is performed in predictions. If the predicted results are much closer to the actual values, the loss will be minimum, while if the results are far away from the original values, then the loss value will be the maximum.

In this section, an experiment is conducted using three loss functions to figure out the most suitable one for the application of CP, and they include:

- Logistic, deviance, or cross-entropy loss is the negative log-likelihood of the Bernoulli model. It is the default loss function in the GBM, and it is defined as^48^:
  25 $${\mathrm{L}}_{Logi}\left(\mathrm{y}, \widehat{ y}\right)=-y\mathit{log} (\widehat{ y})+(1-y)\mathit{log} (1-\widehat{ y})$$
- Bernoulli, it can be formulated as follows^46^.
  26 $${\mathrm{L}}_{Bern}\left(\mathrm{y}, \widehat{ y}\right)=\mathrm{log}\left(1+\mathrm{exp}\left(-2y.\widehat{ y}\right)\right),$$
- Exponential is also used in the Adaboost algorithm, and it can be defined as^48^:
  27 $${\mathrm{L}}_{Ada}\left(\mathrm{y}, \widehat{ y}\right)=\mathrm{exp}\left(-y.\widehat{ y}\right),$$
  where $y$ is a binary class indicator, either 0 or 1, and $\widehat{y}$  is the probability of class 1, while $1-\widehat{ y}$ is the probability of class 0.

**Figure 3:**
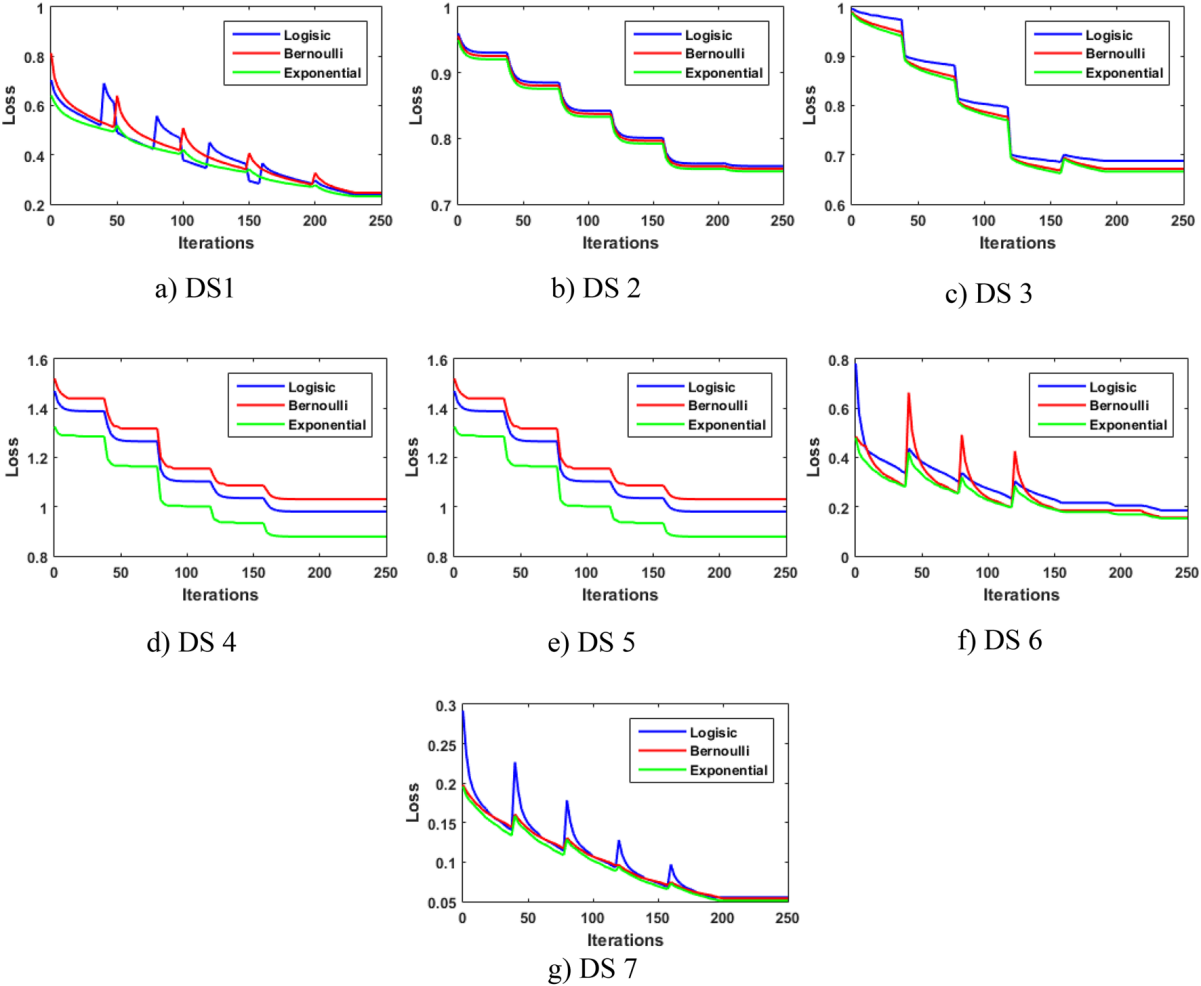
Comparative analysis of loss functions behavior on all the datasets in CP-EGBM framework.

Figure 3 plots the behavior of the loss functions over the defined number of iterations on all the DSs using the developed CP-EGBM. It can be seen in Fig. 3 that the exponential loss function obtains a smaller loss value on all the DSs. This can be explained by exponentially effectively contrasting misclassified data points much more, enabling the CP-EGBM to capture outlying data points much earlier than the logistic and Bernoulli functions. The results from this experiment confirm that the exponential loss function is more suitable than the other two competitor loss functions for the application of CP.

### Hyper-parameter setting

**Figure 4:**
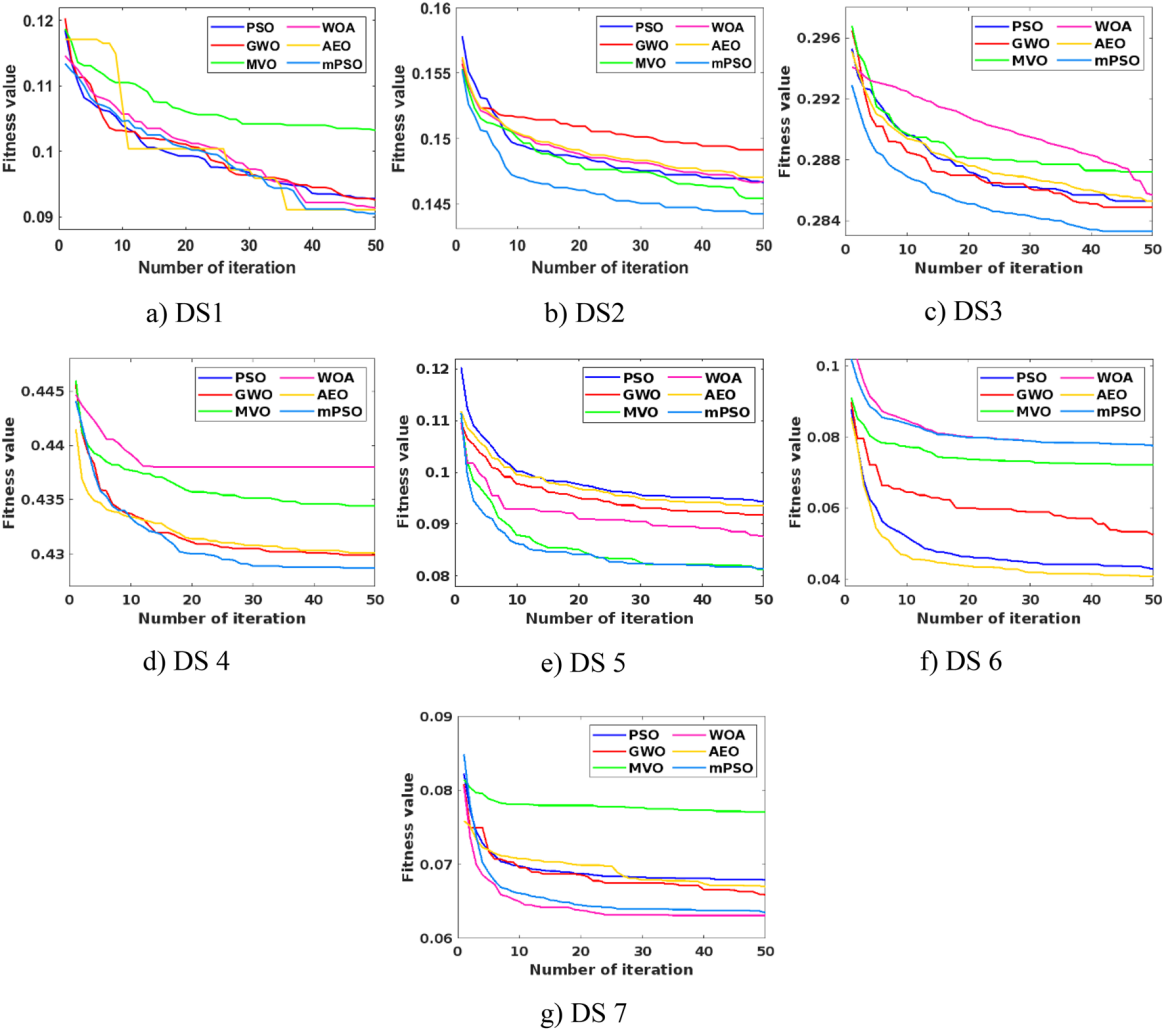
Comparison of convergence behavior of proposed mPSO and other MH algorithms on all the datasets for optimizing CP-EGBM model.

To better understand the behavior of the introduced mPSO, convergence curves are generated over 50 iterations on the x-axis and fitness values on the y-axis, as shown in Fig. 4. A wide range of MH methods introduced in the literature can be used for hyper-parameters tuning. However, the mPSO is compared with Multi-Verse Optimizer (MVO)^49^, Whale Optimization Algorithm (WOA)^50^, Gray Wolf Optimizer (GWO)^51^, PSO^43^, and AEO^44^. For all the methods, the population size is set to 20 and the maximum iterations equal 50. Each is run 20 times, and these settings are selected after empirically studying them. From Fig. 4, the convergence speed of the mPSO is faster than the other MH methods in five out of seven datasets, as it stabilizes to shallow fitness values in fewer iterations. Overall, the suggested improvement in the PSO leads to better convergence attributes and less computation time, making mPSO more suitable for tuning the CP-EGBM model's hyper-parameters.

Several hyper-parameters need to be initialized in the developed CP-EGBM. The mPSO method is used to optimize them. The hyper-parameter settings and the optimized information for each dataset are listed in Tables 2 and 3, respectively.

**Table 2:** Optimization hyper-parameters of different model for tuning the developed CP-EGBM.

Model	Function	Default value	Search space
SVM_RBF_	$\mathrm{C}$	1	LB: 1E−1, UB: 1E
	Mapping of the feature space ($\gamma$)	1/(#features)	LB: 1E−4, UB:1E4
GBM	Number of estimators	100	LB: 100, UB:3000
	Learning rate	0.1	LB: 1E−3, UB:1
	Maximum depth of DTs	3	LB: 1, UB: 10
	Minimum samples for split	2	LB: 2, UB: 10
	Maximum features	Sqrt(#features)	LB: 1, UB: #features
	Sub-sample	1	LB: 0.5, UB:1

*LB* lower boundary, *UB* upper boundary.

**Table 3:** Hyper-parameters of different models in CP-EGBM optimized by mPSO for all the datasets.

Model	Function	DS 1	DS 2	DS 3	DS 4	DS 5	DS 6	DS 7
SVM_RBF_	Regularization ($\mathrm{C}$)	100	156	50	65	25	120	87
	Kernel coefficient ($\gamma$)	0.213	0.302	0.030	0.001	0.003	0.203	0.137
GBM	Number of estimators	315	503	223	418	438	250	305
	Learning rate	0.093	0.103	0.132	0.312	0.034	0.001	0.003
	Max. depth of DTs	5	5	4	6	6	7	6
	Min. samples for split	5	8	6	10	7	8	9
	Max. features	8	12	25	40	8	8	6
	Sub-sample	1	0.82	0.90	0.95	0.83	0.97	0.83

### Experimental results and discussion

The results of the GBM, SVM_RBF,_ and the developed CP-EGBM models using evaluation metrics, Receiver Operating Characteristic (ROC), Statistical test, and model stability are discussed in this section. Also, a comparison between the CP-EGBM and other used models in recent works is provided.

#### Performance results

The performance assessment of the GBM alone, SVM_RBF_ alone, and the developed CP-EGBM models on the datasets is carried out in this section. After applying tenfold-CV and fine-tuning the model's hyper-parameters using the mPSO, the average results are computed and recorded in Tables 4, 5, and 6, respectively.

**Table 4:** Performance evaluation of the GBM alone on all the datasets.

Dataset	*AC*	*R*	*F*	*AUC*
DS 1	0.9401	0.7931	0.8439	0.8246
DS 2	0.8677	0.8514	0.8200	0.8062
DS 3	0.6737	0.6528	0.6813	0.7062
DS 4	0.5631	0.6063	0.5902	0.6160
DS 5	0.9352	0.7825	0.8413	0.8187
DS 6	0.9520	0.8747	0.8672	0.8774
DS 7	0.9520	0.7747	0.8150	0.8274

**Table 5:** Performance evaluation of SVM_RBF_ alone on all the datasets.

Dataset	*AC*	*R*	*F*	*AUC*
DS 1	0.8799	0.8050	0.8410	0.8462
DS 2	0.8376	0.6743	0.7394	0.7971
DS 3	0.6836	0.6889	0.7009	0.7070
DS 4	0.6157	0.6157	0.6146	0.6261
DS 5	0.8821	0.8050	0.8407	0.8462
DS 6	0.8711	0.8749	0.9004	0.8875
DS 7	0.8711	0.7549	0.8202	0.8275

**Table 6:** Performance evaluation of the optimized CP-EGBM on all the datasets.

Dataset	*AC*	*R*	*F*	*AUC*
DS 1	0.9623	0.9121	0.8698	0.8579
DS 2	0.8649	0.8456	0.8211	0.8991
DS 3	0.6949	0.7138	0.7044	0.7091
DS 4	0.6250	0.6298	0.6287	0.6329
DS 5	0.9482	0.9175	0.8727	0.8599
DS 6	0.9779	0.9033	0.9152	0.9273
DS 7	0.9520	0.9275	0.8609	0.8473

**Figure 5:**
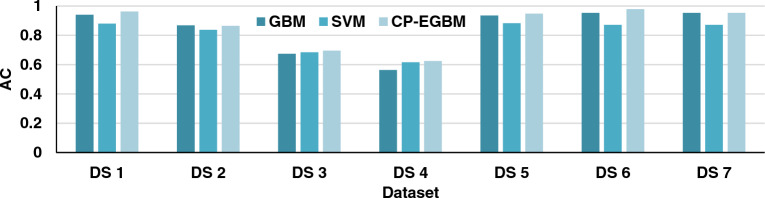
Comparative accuracy analysis of GBM, SVM_RBF_, and CP-EGBM on all the datasets.

**Figure 6:**
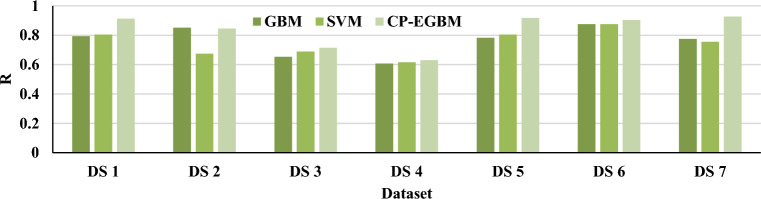
Comparative recall analysis of GBM, SBM_RBF_, and CP-EGBM on all the datasets.

**Figure 7:**
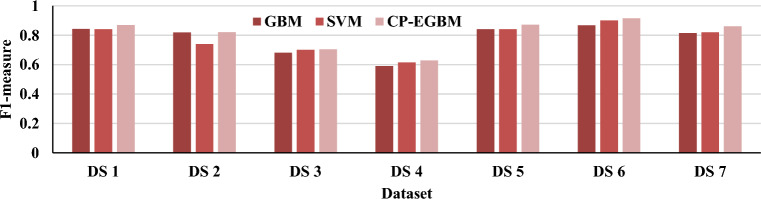
Comparative F1-measure analysis of the GBM, SVM_RBF_, and CP-EGBM on all the datasets.

**Figure 8:**
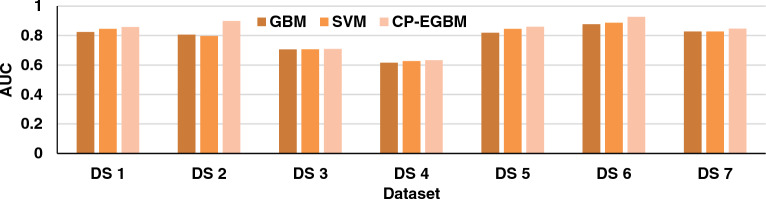
Comparative AUC analysis of the GBM, SVM_RBF_, and CP-EGBM on all the datasets.

The results in Tables 3, 4, and 5 show that the developed CP-EGBM performs better than the other models on all the datasets for individual evaluation metrics. Figures 5, 6, 7, and 8 show the models' performance on all the datasets. These figures reveal that the CP-EGBM has accomplished effective outcomes compared to GBM and SVM_RBF_. For instance, in dataset 6, the CP-EGBM obtained an accuracy of 97.79%, a recall of 90.33%, an F1-measure of 91.52%, and an AUC of 92.73%. The results in Tables 3, 4, 5 and Figs. 5, 6, 7, 8 confirm the superiority of CP-EGBM compared to other models.

For DS 1, SVM has relatively good accuracy (0.8799) and F1-measure (0.8410), while GBM has high better accuracy (0.9401) and F1-measure (0.8439). The developed CP-EGBM outperforms both with the highest accuracy (0.9623) and F1-measure (0.8698). For DS 2, SVM alone has moderate performance with accuracy (0.8376) and F1-measure (0.7394), GBM provides accuracy (0.8677) and F1-measure (0.8200), while CP-EGBM provides relatively high accuracy (0.8649) and F1-measure (0.8211). In DS 3, GBM shows the worst performance in accuracy (0.6737) and F1-measure (0.6813). At the same time, CP-EGBM has the best accuracy (0.6949) and F1-measure (0.7044). Similarly, for DS 4 GBM has the lowest accuracy (0.5631) and F1-measure (0.5902) and CP-EGBM shows high performance with accuracy (0.6250) and F1-measure (0.6287). On the other hand, GBM performs better than SVM for DS5. CP-EGBM outperforms both with high accuracy (0.9482) and F1-measure (0.8727). Similar observations can be made for DS 6 with an outstanding performance of CP-EGBM by providing very high accuracy (0.9779) and F1-measure (0.9152). For DS 7, both GBM and CP-EGBM provide the same accuracy but later have higher F1-measure than earlier.

Overall, CP-EGBM consistently outperforms both GBM and SVM across most of the datasets in terms of accuracy, F1-measure, and AUC. However, GBM and SVM show competitive performance, achieving high accuracy and F1-measure on some datasets but lower performance on others.

#### ROC curve

**Figure 9:**
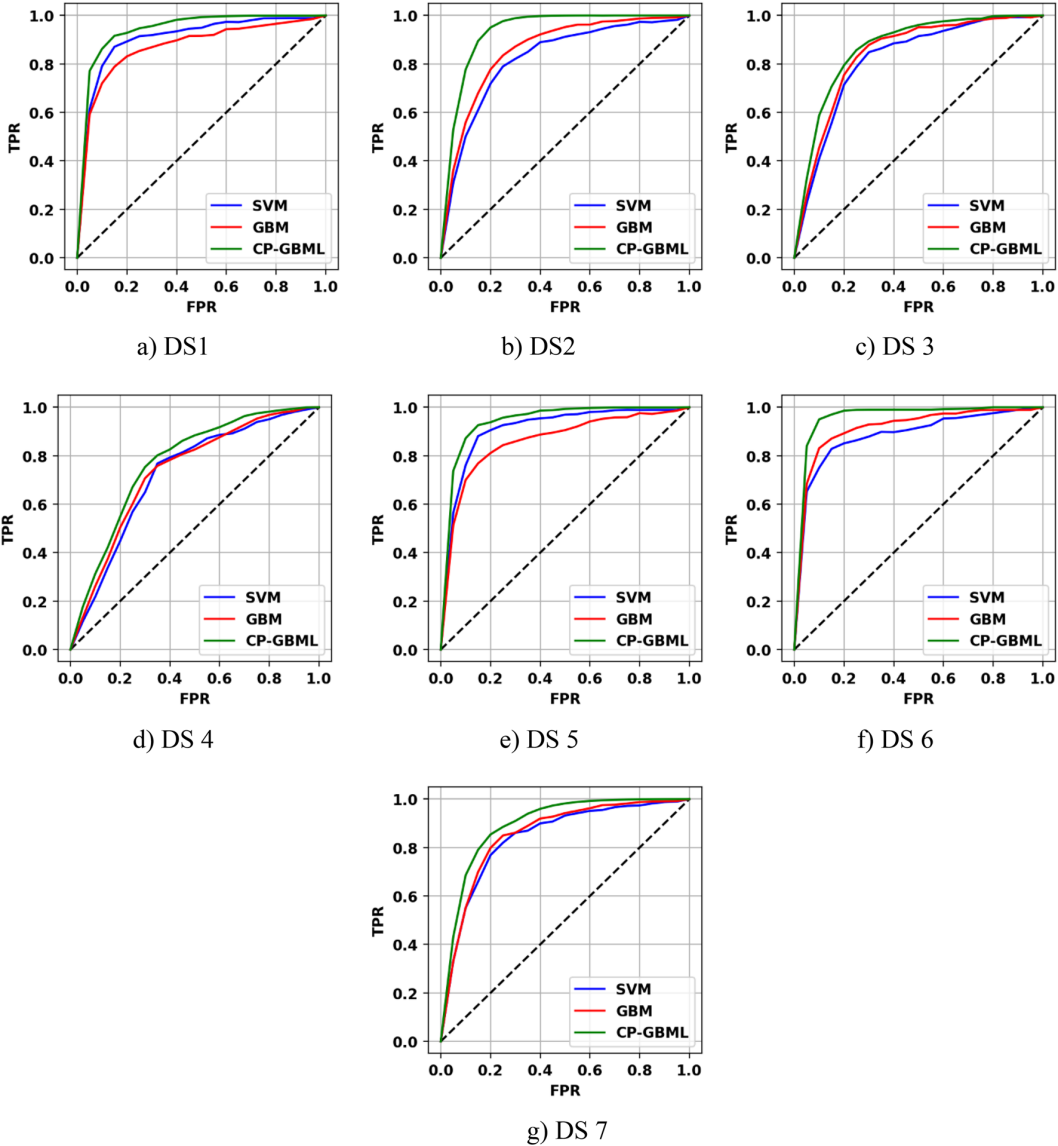
ROC-based performance comparison of GBM, SVM_RBF_, and CP-EGBM for all the datasets.

The ROC curve computes model performance by changing the confidence level of the model score to get distinct values of the True-Positive Rate (TPR) and False Positive Rate (FPR), as illustrated in Fig. 9. As this figure shows, the CP-EGBM curves dominate the GBM and SVM_RBF_ models in all points on all the considered datasets, which indicates the suitability of the developed CP-EGBM.

#### Statistical test and model's stability

**Figure 10:**
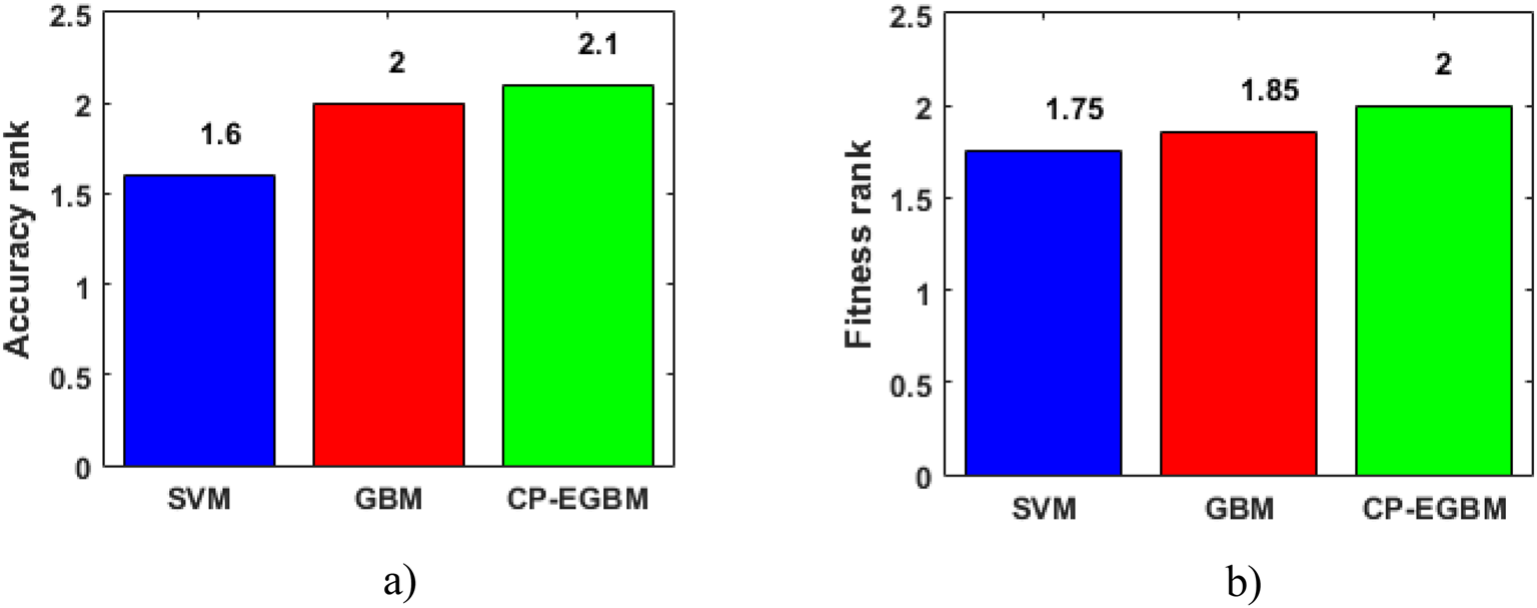
Friedman ranks test for statistical comparison of (**a**) accuracy, (**b**) fitness values of different model.

The developed CP-EGBM is selected as the control model in the Friedman ranks test, as shown in Fig. 10. In this figure, CP-EGBM gets the highest accuracy (Fig. 10a) and fitness values ranks (Fig. 10b), followed by GBM as the second and the SVMRBF ranked last. Therefore, this work concludes that the CP-EGBM is significantly better than the other models for CP.

The relative stability results associated with the standard deviation (Std) of the developed CP-EGBM and the other models are also calculated and provided in Table 7. According to the results in Table 6, the developed CP-EGBM model achieved the smallest Std values compared to the GBM and SVM_RBF_ models on all the datasets. This reflects the stability and robustness of the developed CP-EGBM for applying CP.

**Table 7:** Comparison of stability and robustness of all models using Std values for all the datasets.

Dataset	Measure	Model		
		GBM	SVM_RBF_	CP-EGBM
DS 1	Std	0.0137	0.0111	**0.0091**
DS 2	Std	0.0678	0.0401	**0.0204**
DS 3	Std	0.1025	0.0913	**0.0467**
DS 4	Std	0.1008	0.0925	**0.0381**
DS 5	Std	0.0123	0.0102	**0.0086**
DS 6	Std	0.0106	0.0097	**0.0055**
DS 7	Std	0.0107	0.0094	**0.0078**

Significant values are in bold.

#### Performance comparison with existing models

Several studies have recently used ML models to predict customer churn in the telecom sector. A comparison between the developed CP-EGBM and other studies for CP is given in Table 8. We can see in Table 7 that the studies utilized DS 1, DS 2, and DS 5 to evaluate ML models used in their works. Therefore, we can use the same DSs to compare the performance of the CP-EGBM with them. As per the results in Table 8, the developed CP-EGBM model has great potential to predict customer churn in terms of accuracy and F1-measure with better prediction results than the existing models.

**Table 8:** Comparison between the existing models and the proposed CP-EGBM model in terms of accuracy and F1-measure on DS 1, DS 2, and DS 5.

Author (s)	DS 1		DS 2		DS 5	
	*AC*	*F*	*AC*	*F*	*AC*	*F*
Sabbeh (2018)^22^	0.9600	–	–	–	–	–
Sandhya et al*.* (2021)^23^	0.9550	0.8210	–	–	–	–
Kimura (2022)^24^	–	–	0.7710	0.613	–	–
Zhu and Liu (2021)^25^	–	–	0.7998	–	–	–
Kanwal et al*.* (2021)^26^	0.9300	0.8110	–	–	–	–
Bilal et al*.* (2022)^27^	0.9243	0.7181	–	–	0.9470	0.8063
Karuppaiah and Gopalan (2021)^28^	0.8900	–	–	–	–	
Rabbah et al*.* (2022)^29^,	–	–	0.8350	0.8190	–	–
Karamollaoglu et al*.*^30^	0.9540	0.9440	–	–	0.7900	0.8630
Akbar and Apriono (2023)^32^,	–	–	0.8156	0.7476	–	–
Developed CP-EGBM	**0.9623**	**0.8698**	**0.8649**	**0.8211**	**0.9482**	**0.8727**

Significant values are in bold.

The proposed framework uses MH algorithms for feature selection and hyper-parameter tuning. Although MH algorithms have shown effectiveness in many domains, they also have certain limitations. MH algorithms may converge prematurely to get stuck in a local optimum or fail to explore the search space adequately. MH algorithms require many iterations and evaluations of objective functions, which can be computationally expensive for complex problems. Several control parameters need to be set appropriately to achieve good performance. The search process becomes more challenging in high-dimensional spaces, and MH algorithms may struggle to explore and exploit the search space effectively. Despite these limitations, MH algorithms remain valuable tools for solving complex optimization problems.

## Conclusion and future works

The telecom sector has accumulated a massive amount of customer information during its development, and on the other hand, the widespread data warehouses technology and application make it possible to gain insight into historical customer data. Therefore, it has become clear to managers in this sector that customer information can be used to create prediction models to contain customer churn and risk. A CP-EGBM model is developed to provide an efficient prediction model for the application of CP. The CP-EGBM model uses SVM as a base learner and DTs as weak learners in the GBM's structure. Moreover, a modified version of PSO, mPSO, is introduced to optimize the CP-EGBM model's hyper-parameters by injecting the AEO consumption operator into the PSO's structure. The CP-EGBM is assessed using seven CP datasets. The experimental results and statistical test analysis show higher efficacy of the CP-EGBM model than GBM, SVM, and reported models in the literature. The results confirm the ability of the developed model for CP in the telecommunications sector. In the future, we will use the developed CP-EGBM to address other CP-related problems in e-commerce, businesses, and online shopping applications. Also, will deploy CP-EGBM on more datasets to make its results more robust. We will also apply the suggested mPSO method to solve feature selection problems in different fields, such as sentiment analysis and the Internet of Things. In sentimental analysis, each sentence is represented using a high dimensional sparse vector due to tokenization in natural language processing. Similarly, in IoT applications, inputs are represented using high-dimensional vectors because of the large number of sensing nodes. In these applications, mPSO can be used to reduce feature dimensionality, reducing the computational complexity of such systems.

## Data Availability

The datasets used and/or analyzed during the current study are available from the corresponding author upon reasonable request.

## Abbreviations

CP: Churn prediction
EGBM: Enhanced gradient boosting machine
ACO: Ant colony optimization
ACO-RSA: Ant colony optimization-reptile search algorithm
AEO: Artificial ecosystem optimization
AUC-ROC: Area under the receiver operating characteristic curve
CNN: Convolutional neural network
CP-EGBM: Enhanced gradient boosting machine for churn prediction
CRM: Customer relationship management
CV: Cross validation
DT: Decision tree
FN: Flase negative
FP: False positive
GBM: Gradient boosting machine
GBT: Gradient boosted tree
GWO: Gray wolf optimizer
HEOMGA: Heterogeneous euclidean-overlap metric genetic algorithm
KNN: K-nearest neighbors
LR: Logistic regression
MH: Meta-heuristic
ML: Machine learning
mPSO: Modified particle swarm optimization
MVO: Multi-verse optimizer
NB: Naive Bayes
PSO: Particle swarm optimization
RBF: Radial basis functions
RF: Random forest
SMOTE: Synthetic minority oversampling technique
SVM: Support vector machine
SVM_RBF_: Support vector machine with RBF kernel
TN: True negative
TP: True positive
WOA: Whale optimization algorithm
XGBoost: Extreme gradient boosting
